# Treatment of Bacterial Vaginosis: A Multicenter, Double-Blind, Double-Dummy, Randomised Phase III Study Comparing Secnidazole and Metronidazole

**DOI:** 10.1155/2010/705692

**Published:** 2010-09-15

**Authors:** Jean-Marc Bohbot, Eric Vicaut, Didier Fagnen, Michel Brauman

**Affiliations:** ^1^Institut Alfred Fournier, 25, Boulevard Saint-Jacques, 75014 Paris, France; ^2^Hôpital Fernand Widal, 200 rue du Fbg Street Denis, 75010 Paris, France; ^3^IPRAD 174, quai de Jemmapes, 75010 Paris, France

## Abstract

*Objective*. Multiple-dose metronidazole oral therapy is currently the reference treatment for bacterial vaginosis (BV). This double-blind, double-dummy, noninferiority study compared the efficacy of secnidazole, another nitroimidazole with pharmacokinetics allowing a single dose regimen, to this standard treatment. *Methods*. A total of 577 patients were randomized to receive metronidazole (500 mg, b.i.d for seven days) or secnidazole (2 g, once). Therapeutic cure at D28 was defined as the resolution of vaginal discharge, positive KOH whiff test, vaginal pH >4.5 and Nugent score >7 on Gram-stained vaginal fluid. *Results*. According to this primary endpoint, the single-dose secnidazole regimen was shown to be at least as effective as the multiple-dose metronidazole regimen (60.1 % cured women vs 59.5% , 95% confidence interval with a noninferiority margin of 10%: [−0.082; 0.0094]). Safety profiles were comparable in both groups. *Conclusion*. The secnidazole regimen studied represents an effective, convenient therapeutic alternative that clinicians should consider in routine practice.

## 1. Introduction

Bacterial vaginosis (BV) is a common cause of vaginal discharge, occurring in up to 30% of women [1]. It is associated with a complex change in vaginal flora including a decrease in normal *Lactobacillus* spp., and an increase in *Gardnerella vaginalis* and anaerobes [1, 2]. 

Essentially characterized by a fishy-smelling, thin, greyish vaginal discharge [2], BV is clinically diagnosed using the Amsel criteria, including four parameters: presence of the typical discharge, a vaginal pH > 4.5, a positive whiff test (amine odour after adding 10% potassium hydroxide to vaginal fluid) and the presence of “clue cells” (epithelial cells with adhering bacteria) on microscopic examination. At least three out of these four criteria have to be present [2]. The diagnosis may be confirmed by a Nugent score equal or higher than seven on bacteriological analysis of vaginal samples. 

Besides being unpleasant for patients when symptoms of discharge and odour occur, BV is associated with an increased risk of several pathological gynaecological conditions as well as major adverse outcomes during pregnancy. It is estimated that BV is associated with a twofold increased risk of preterm birth (odds ratio: 2.4; 95% CI: 1,2–4,8) and a sixfold increased risk of miscarriage (odds ratio: 6.6; 95% CI: 2.1–20.9) [3]. 

Although the pathogenesis of BV is still not clearly understood and the aetiological role played by the organisms replacing the normal aerobic vaginal flora remains uncertain, antibiotics with good activity against anaerobes but that do not affect *Lactobacillus* spp. represent the mainstay of BV therapy [2]. Metronidazole, administered either orally or topically according to multiple-dose regimens, has long been established as a standard treatment of BV [4–6]. However, a drawback of these regimens is the necessity to administer them for several days, potentially diminishing compliance with a risk of incomplete cure and recurrence of BV [7].

Secnidazole is a new second-generation 5-nitroimidazole product with a broad spectrum of activity against anaerobic bacteria and a longer half-life than metronidazole, making it suitable for single-dose therapy, and therefore potentially offers an advantage over multiple-dose metronidazole regimens. Several studies have consistently suggested its clinical benefits for the treatment of BV [8–10]. However, their methodology was somewhat questionable and there was a need to investigate the efficacy of secnidazole in a well-designed study satisfying the most recent guidelines like these issued by the Food and Drug Administration (FDA).

## 2. Materials and Methods

This was a national, multicentre, prospective, randomised, comparative, double-blind, double-dummy, Phase III, noninferiority study comparing the efficacy of secnidazole *versus* metronidazole in patients with bacterial vaginosis. Nonpregnant women aged 18–65 years with clinical signs of bacterial vaginosis, and from whom a vaginal sample had been collected at the preinclusion visit, were eligible for enrolment. The clinical diagnosis of BV was established on the basis of the following three Amsel criteria: a homogeneous, thin, greyish-white vaginal discharge, positive potassium hydroxide whiff test results, and a vaginal pH above 4.5 [11]. The diagnosis of BV was later confirmed by a Nugent score above seven on bacteriological analysis of the preinclusion vaginal samples. Patients were excluded if they had received antibiotic or antifungal drugs within the past 14 days. The study was approved by the Ethics Committee of Kremlin Bicêtre Hospital (France) and all women gave written informed consent before starting the study.

### 2.1. Study Design

After baseline screening, patients were randomised to metronidazole (reference treatment) or secnidazole (study treatment) in a 1 : 1 ratio. Randomisation was stratified by clinical centre; the randomisation list was computer-generated (SAS software) with block sizes of four (two secnidazole, two metronidazole). Investigators and patients were blind to study treatments. 

After randomisation, patients received either a single 2 g dose of secnidazole or the reference treatment, that is, a seven-day course of 500 mg metronidazole twice daily. In view of the difference in pharmaceutical form and administration regimen between metronidazole (capsules, multiple-dose) and secnidazole (sachet, single-dose), the trial was designed as a double-dummy study. According to the randomisation schedule, patients received either the study treatment and the placebo of the reference treatment or the reference treatment and the placebo of the study medication (one sachet on Day 1, and two capsules per day from Day 1 to Day 7). The patients attended two followup visits, at day 14 (D14) ± 2 days and at day 28 (D28) ± 2 days. At each visit, a clinical examination was performed and vaginal samples were taken.

### 2.2. Endpoints

In accordance with the current FDA guidelines, the primary efficacy endpoint was the therapeutic success, that is, a composite of clinical and bacteriological cure, at D28 [12]. Clinical cure was defined as the normalisation of the three Amsel criteria and bacteriological cure was defined as a Nugent score lower or equal than three. The secondary efficacy criteria were therapeutic success at D14, clinical cure at D14 and D28, bacteriological cure at D14 and D28, mean time to symptom disappearance, and safety. Patients had to complete a daily questionnaire from D1 to D14, recording the intensity of any vaginal discharge (severe, moderate or absent) and the existence of an unpleasant odour (yes/no). Safety was assessed on the basis of adverse events reported.

### 2.3. Populations

The primary efficacy endpoint was analysed on the intention-to-treat (ITT) population, including all randomised patients who received at least one dose of the treatment, on the modified ITT (mITT) population defined as all the patients of the ITT population in whom the diagnosis of BV was confirmed after bacteriological examination, and on the per protocol (PP) population comprising all patients included in the mITT population who completed the study protocol without any major deviation and were evaluable at all study visits. The main population was mITT population, but consistent results between mITT and PP populations were expected due to the noninferiority design of the study. Patients not evaluable at D28 were reported as “therapeutic failures” in the ITT and mITT populations. Safety analyses were performed on the ITT population.

### 2.4. Sample Size and Statistical Analysis

The study was designed with ≥90% power to test the hypothesis that a single dose of secnidazole is noninferior to a seven-day course of metronidazole. According to published studies, the cure rate with the reference treatment was estimated to be approximately 87% [13]. Assuming this cure rate, a noninferiority margin fixed at 10% and a similar dropout rate in the two groups, a sample size of 432 patients (216 per group) was needed to determine noninferiority with a power of 80% and an alpha error of 2.5% (one-sided test). Postulating that 25% of recruited patients would not be evaluable for the primary criteria, the targeted recruitment was 287 patients per group. 

Noninferiority was tested using the confidence interval (CI) approach: the primary and secondary efficacy endpoints were analysed by calculation of the bilateral (two-sided) 95% CI of the difference in rate “Secnidazole group-Metronidazole group”. The lower limit of the CI was compared to the −10% limit of noninferiority. The mean time to symptom disappearance was compared between the two groups using the log-rank test. Safety data were analysed using Chi-square tests (Pearson's or Fisher's).

## 3. Results and Discussion

### 3.1. Results

**Figure 1:**
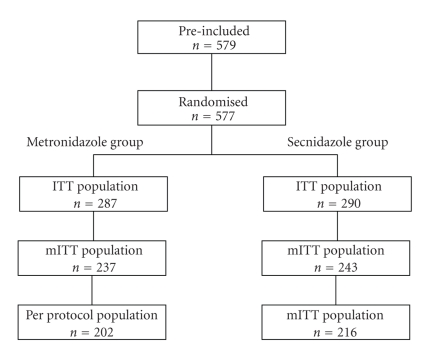
Treatment groups and populations.

The study was performed between March 2007 and July 2008 at 27 sites in France. A total of 577 women (mean age: 36 years in both groups) were enrolled and randomised to receive secnidazole (ITT, *n* = 287) or metronidazole (ITT, *n* = 290) (Figure 1). Approximately 28% of patients (secnidazole: 27.2%; metronidazole: 28.6%) had experienced at least one episode of BV during the two years preceding inclusion. After bacteriological examination, the diagnosis of BV was confirmed in 237 patients (81.7%) in the metronidazole group and 243 patients (84.7%) in the secnidazole group (mITT population). During the study, 16 premature withdrawals (metronidazole, *n* = 12; secnidazole, *n* = 4; *P* = .045) were reported, the main reasons cited being “personal convenience” (metronidazole, *n* = 4; secnidazole, *n* = 3) and “lost to followup” (metronidazole, *n* = 3). At the end of the study, major deviations from the protocol, most frequently nonrespect of scheduled visits, nonevaluability of the primary efficacy endpoint and concomitant antibiotic treatment, were reported for 35 subjects in the metronidazole group and 27 subjects in the secnidazole group (Table 1). “Nonrespect of compliance” was reported for seven patients in the metronidazole group and four patients in the secnidazole group. Overall, 202 patients in the metronidazole group and 216 patients in the secnidazole group were included in the PP population.

#### 3.1.1. Primary Efficacy Endpoint: Therapeutic Success at D28

In the mITT population, therapeutic success (both clinical and bacteriological cure) at D28 was achieved in similar percentages of patients in both groups: 59.5% (141/237) in the metronidazole group and 60.1% (146/243) in the secnidazole group (Table 2). The lower limit of the 95% confidence interval of the difference “secnidazole-metronidazole” was above −10% ([−0.082; 0.094]), confirming the noninferiority of secnidazole compared to metronidazole. The rates of therapeutic success in the PP and ITT populations confirmed the noninferiority of secnidazole compared to metronidazole (Table 2).

#### 3.1.2. Therapeutic Success at D14

At D14, therapeutic success in the mITT population was observed in 66.2% (157/237) of patients in the metronidazole group* versus* 65% (158/243) of patients in the secnidazole group (Table 3). The noninferiority of secnidazole was confirmed by the limits of the 95% CI for the difference “secnidazole-metronidazole” (95% CI: [−0.097; 0.073]). In the PP population, the percentages of patients achieving therapeutic success were similar in the two treatment groups (Table 3), corroborating the noninferiority of secnidazole. In the ITT population, the lower limit of the 95% CI was slightly below −10%, but the difference between the groups was not statistically significant.

#### 3.1.3. Clinical and Bacteriological Cures Assessed Separately

At D28, clinical cure was achieved in 77% of patients in the secnidazole group and bacteriological cure in 70.3%, higher percentages than the therapeutic success rate (≈60%), as some patients achieved clinical cure but not bacteriological cure and vice versa. Similar results were obtained with metronidazole (Table 4). Considering clinical cure not only with bacteriological cure but also with bacteriological improvement (Nugent score between three and seven), the percentage of responding patients increased to around 70% in both groups (Table 5). Analysis of the PP and ITT populations gave similar results, with little difference between the secnidazole and metronidazole groups (data not shown).

#### 3.1.4. Mean Time to Symptom Disappearance

Among the patients of the mITT population completing the self-assessment diary, more than three-quarters reported the disappearance of BV symptoms, within a mean of 7.12 days in the metronidazole group and 6.83 days in the secnidazole group (Table 6). Similar results were observed in the PP and ITT populations (data not shown).

#### 3.1.5. Safety

Safety was evaluated in all randomised patients who took at least one dose of the study treatment. In the two treatment groups, a similar proportion of patients experienced at least one adverse event (AE): 109 (38%) in the metronidazole group and 113 (39%) in the secnidazole group. No differences were observed in the frequencies of AE classified by Organ System, with the exception of headaches, more frequent, although rare, in the secnidazole group (*n* = 10 versus *n* = 4 in the metronidazole group). The difference in the rate of patients reporting an expected AE between the metronidazole group (*n* = 27, 9.4%) and the secnidazole group (*n* = 16, 5.5%) was at the limit of statistical significance testing. The percentages of subjects reporting at least one drug-related AE were similar in the two treatment groups: 22.7% (*n* = 65) in the metronidazole group and 22.4% (*n* = 65) in the secnidazole group, most of these AE being mild in intensity (66.2% in the metronidazole group and 67.7% in the secnidazole group) and associated with complete recovery by the end of the followup period (28 days) (49.2% of patients in the metronidazole group and 53.9% of patients in the secnidazole group). The most frequent unresolved events (vaginitis and abnormal genital discharge) could be considered as treatment failure.

### 3.2. Discussion

This large, randomised, double-blind, double-dummy Phase III clinical trial designed according to the most recent FDA guidance [12] confirmed the efficacy and safety of a single-dose regimen of secnidazole compared to the standard multiple-dose metronidazole regimen. 

In all patient populations (ITT, mITT, and PP), around 60% of patients in both treatment groups achieved both bacteriological and clinical cure at D28. The time to symptom disappearance was also similar in both groups. The observed therapeutic success rate was lower than those reported in previous trials investigating oral metronidazole in this indication. In a systematic review of metronidazole treatment of BV published in 1992, the four studies evaluating metronidazole 500 mg bid for seven days reported an initial cure rate within four weeks ranging between 83% and 97% [13]. The recently published Cochrane Review assessing the effectiveness of antimicrobial agents used to treat BV in nonpregnant women, excluded these four studies, as the methods used for BV diagnosis were deemed doubtful, but selected seven other similar trials, mostly performed in the late 1980s and early 1990s [2, 14–20]. Once again, the cure rates reported, ranging between 78% and 96%, were substantially higher than that recorded in our study (60%). 

Several explanations may be postulated for this discrepancy. Firstly, whereas in our study, diagnosis and cure were defined according to the stringent definitions given in the FDA guidance [12] taking into account both bacteriological and clinical criteria, most previous studies used less rigorous endpoints often based on clinical criteria only. Interestingly, the clinical cure rate achieved at D28 (secondary endpoint) in our metronidazole group of patients (77%) is close to the rates reported in these previous studies. Secondly, these older studies, some being open-label, included fewer patients. Finally, therapeutic cure was sometimes assessed after too short a time posttreatment, and this is a crucial limitation since evaluation of BV before one month of treatment is known to be inaccurate [21]. 

Considering only the two previous randomised, double-blind, controlled studies including at least 100 patients, “clinical cure rates” reported at D28 after treatment with metronidazole were lower [17, 19]. Both these studies compared oral metronidazole (500 mg twice a day for seven days) versus vaginal clindamycin in approximately 400 patients. In one study, cure of BV defined as a pH ≤ 4.5, absence of amine odour after addition of potassium hydroxide, and absence of clue cells, was observed in 54% of patients in the metronidazole group at one month posttherapy [17]. Cure or improvement (requiring two of the criteria defining cure) was achieved by 78% of patients. In the other study, the overall cure rate determined on the basis of the absence of clue cells and of an amine odour reached 76.3% at the second followup visit scheduled between 28 and 42 days after the start of treatment [19]. Interestingly, these rates are of the same order of magnitude as the clinical cure rate achieved at D28 (secondary endpoint) in our metronidazole group: 77%.

Similarly, a recent review recalculating the four-week cure rate for oral metronidazole based on the results of published placebo-controlled studies, quoted an anticipated average cure rate of 66%, which is in accordance with our therapeutic success rate [22]. 

In the previous studies assessing the efficacy of a single oral 2 g dose of secnidazole for the treatment of BV, the reported cure rates (72% and 93%) were also higher than the therapeutic success rate observed at D28 in our secnidazole-treated patients (≈60%), probably for the same methodological reasons [8, 10].

The secondary endpoint analysis showed that therapeutic success rates at D14 were slightly superior to those observed at D28, both in the secnidazole group (62.4% versus 58.3%) and in the metronidazole group (65.2% versus 57.8%), testifying either relapse or recurrence, probably due to the persistence of the original imbalance in vaginal flora. The difference in success rates at D14 and D28 observed was of the same order within each treatment group. Available data indicate that when BV reappears, it is more likely to represent reactivation rather than a new infection [22]. This result highlights the relevance of assessing treatment efficacy at D28, as recommended in the FDA guidelines. 

Secnidazole has been extensively used for the past 20 years to treat various parasitic diseases, including trichomoniasis, and its good safety profile is well established. Neither the preclinical toxicity studies nor the accumulated postmarketing experience in its approved indications gave evidence of a risk of adverse events with secnidazole during pregnancy. This study confirmed that secnidazole is well-tolerated, adverse events recorded being mild in severity and predominantly those known to be associated with imidazole derivatives as a whole. The relationship of adverse events to secnidazole was difficult to evaluate in this study, as the occurrence of adverse events was evaluated three weeks after the administration of a single dose of the drug.

## 4. Conclusion

In conclusion, these results are important to the extent that this randomised, double-blind, double-dummy clinical trial is the first one to assess the efficacy of the reference treatment with oral metronidazole in a large population of patients with BV following a rigorous methodology which conforms to the recent FDA guidance. Secnidazole was at least as effective as metronidazole with a similar favourable safety profile. With its more convenient posology, that is, a single-dose regimen versus a twice-a-day regimen for seven days with the reference drug, secnidazole represents an attractive therapeutic option that should be considered in routine practice, particularly in women whose likely compliance is doubtful or in women who are asymptomatic and question the necessity of a treatment over several days [22].

## Figures and Tables

**Table 1:** Major deviations leading to the exclusion of patients from the per protocol population.

	Metronidazole	Secnidazole
	*n* = 287	*n* = 290
	% (*n*)	% (*n*)
Nonrespect of scheduled visits	4.9 (14)	3.8 (11)
Nonevaluability of primary efficacy endpoint	4.5 (13)	1.7 (5)
Concomitant antibiotic treatment	3.5 (10)	2.1 (6)
Nonrespect of compliance	2.4 (7)	1.4 (4)
of which missing data on compliance	1.4 (4)	1.4 (4)
Error in randomisation	1.4 (4)	1.4 (4)
Treatment initiated more than 3 days after randomisation	1.0 (3)	0 (0)
Nonrespect of inclusion criteria	0.3 (1)	0.7 (2)
Vaginal treatment	0 (0)	0.7 (2)
Nonrespect of exclusion criteria	0 (0)	0.3 (1)

**Table 2:** Overall therapeutic success at D28 (primary efficacy endpoint).

	Population		
	ITT	mITT	PP
Secnidazole	58.3%	60.1%	63.4%
	(169/290)	(146/243)	(137/216)
Metronidazole	57.8%	59.5%	62.9%
	(166/287)	(141/237)	(127/202)
95% CI Secnidazole-Metronidazole	[−0.076; 0.085]	[−0.082; 0.094]	[−0.087; 0.098]

NS: not significant.

**Table 3:** Overall therapeutic success at D14.

	Population		
	ITT	mITT	PPD14*
Secnidazole	62.4%	65.0%	68.7%
	(191/290)	(158/243)	(147/214)
Metronidazole	65.2%	66.2%	69.0%
	(187/287)	(157/237)	(149/216)
95% CI Secnidazole-Metronidazole	[−0.106; 0.051]	[−0.097; 0.073]	[−0.09; 0.085]

*PPD14 (per protocol population at day 14): patients who were assessable and presented no major deviation from the protocol at day 14 (214 patients in the metronidazole group and 216 patients in the secnidazole group).

**Table 4:** Clinical and bacteriological cures at D14 and D28 in the mITT population.

	Clinical cure (%)		Bacteriological cure (%)	
	D14	D28	D14	D28
Secnidazole	79.7%	77%	77.5%	70.3%
(*n* = 243)	(*n* = 193)	(*n* = 187)	(*n* = 188)	(*n* = 171)
Metronidazole	77.9%	79.3%	77.3%	71.4%
(*n* = 237)	(*n* = 145)	(*n* = 188)	(*n* = 183)	(*n* = 169)
95% CI Secnidazole-Metronidazole	[−0.056; 0.093]	[−0.098; 0.052]	[−0.073; 0.079]	[−0.093; 0.072]

NS: not significant.

**Table 5:** Clinical cure with bacteriological improvement and/or cure at D14 and D28 in the mITT population.

	Therapeutic success (%)		Clinical cure with bacteriological cure	
	(i.e., clinical and bacteriological cure)		or improvement* (%)	
	D14	D28	D14	D28
Secnidazole	65.0%	60.1%	72%	69%
(*n* = 243)	(158/243)	(146/243)	(*n* = 174)	(*n* = 167)
Metronidazole	66.2%	59.5%	72%	67%
(*n* = 237)	(157/237)	(141/237)	(*n* = 171)	(*n* = 158)
95% CI Secnidazole-Metronidazole	[−0.097; 0.073]	[−0.082; 0.094]		

*Bacteriological improvement defined as a nugent score between 3 and 7.

**Table 6:** Mean time to symptom disappearance in the mITT population.

	Patients completing the	Patients reporting	Time to symptom disappearance	
	questionnaire	symptom disappearance	(no. of days)	
	% (*n*)	% (*n*)	Mean ± SD	Median ± SD
Secnidazole	84.4%	82.4%	6.83 ± 0.24*	6
(*n* = 243)	(205)	(169)		
Metronidazole (*n* = 237)	86.9% (206)	79.6% (164)	7.12 ± 0.25*	7

*Difference between treatment groups not statistically significant.
